# Supplementary material for: Identification and Characterization of Rhipicephalus microplus ATAQ Homolog from Haemaphysalis longicornis Ticks and Its Immunogenic Potential as an Anti-Tick Vaccine Candidate Molecule
Source: Microorganisms. 2023 Mar 23;11(4):822. doi: 10.3390/microorganisms11040822 (PMC10145298; doi:10.3390/microorganisms11040822)
Supplement: Supplementary file 1 [file microorganisms-11-00822-s001.zip › Supplementary Materials (Adjou Moumouni et al.)_R2.pdf]

Supplementary Materials

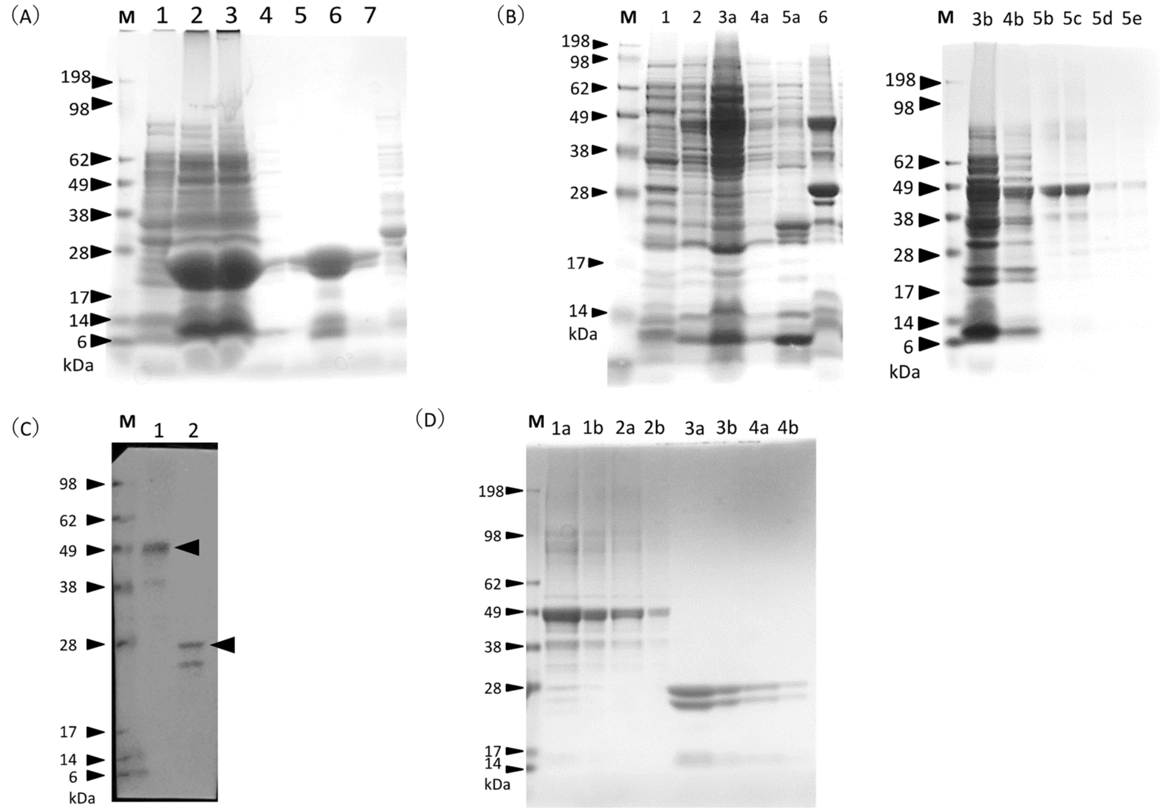

Figure S1. Expression, purification and verification of immune response to recombinant proteins. A) recombinant ProS2-tag (rProS2) expression and purification in Escherichia coli BL21 (DE3). Each protein (10μl) was applied and separated by SDS-PAGE 10%. Lane M: protein marker; Lane 1, a lysate of bacteria carrying rProS2 before IPTG induction; Lane 2, the lysate of bacteria carrying rProS2 after IPTG induction; Lane 3, a flow-through after rProS2 capture; Lane 4, a wash #1 without the captured rProS2; Lane 5, a wash #2 without the captured rProS2; Lane 6, the 1st elution of rProS2 from a Capturem His-Tagged Purification column; Lane 7, the 2nd elution of rProS2 from the Capturem His-Tagged Purification column; Lane 8, debris in 8M urea buffer. B) recombinant ProS2-truncated HlATAQ (rtHlATAQ) expression and purification in *E. coli* BL21 (DE3). Each protein (10μl) was applied and separated by SDS-PAGE 12.5% (Lanes 1, 2, 3a, 4a, 5a, 6) or 10% (Lanes 3b, 4b, 5b, 5c, 5d, 5e). Lane M: protein marker; Lane 1, a lysate of bacteria carrying rtHlATAQ before IPTG induction; Lane 2, the lysate of bacteria carrying rtHlATAQ after IPTG induction; Lane 3a, a flow-through after rtHlATAQ capture; Lane 3b, a flow-through after binding rtHlATAQ with Ni-NTA agarose; Lane 4a, a wash #1 without the captured rtHlATAQ; Lane 4b, a wash #1 without the rtHlATAQ bound with N-NTA agarose; Lane 5a, the 1st elution of rtHlATAQ from a Capturem His-Tagged Purification column; 5b, the 1st elution of rtHlATAQ from Ni-NTA agarose; Lane 5c, the 2nd elution of rtHlATAQ from Ni-NTA agarose; Lane 5d, the 3rd elution of rtHlATAQ from Ni-NTA agarose; Lane 5e, the 4th elution of rtHlATAQ from Ni-NTA agarose; Lane 6, debris dissolved in 8M urea buffer. C) The immune response to rtHlATAQ and rProS2 was verified using a Western blotting. Twenty-nanogram of purified rtHlATAQ (Lane1) and rProS2 (Lane 2) were used in the Western blotting with anti-rPros2 mouse monoclonal antibodies. D) Dialysed rtHlATAQ and rProS2. Lane 1a (5 μl) and 1b (2.5 μl), a mixture of the 2nd elution obtained several purifications (0.704 μg/μl rtHlATAQ); Lane 2a (5 μl) and 2b (2.5 μl), a mixture of the 1st elution obtained several purifications (0.492 μg/μl rtHlATAQ); Lane 3a (5 μl) and 3b (2.5 μl), a mixture of the 1st elution obtained several purifications (0.812 μg/μl rtHlATAQ); Lane 4a (5 μl) and 4b (2.5 μl), a mixture of the 2nd elution obtained several purifications (0.468 μg/μl rtHlATAQ).

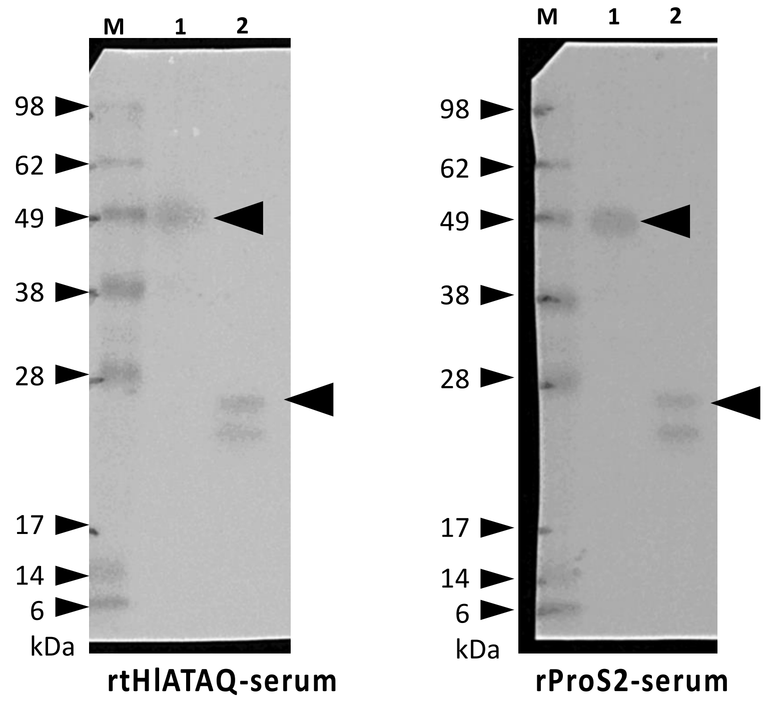

Figure S2. Confirmation of the specificity of the reactivity of rtHlATAQ- and rProS2-immunized rabbit sera against rtHlATAQ and rProS2 proteins. After 11 days of post-boost vaccination, sera were isolated from rtHlATAQ- and rProS2-immunized rabbits. Purified proteins (20 ng in each lane) were electrophoresed in 12.5 SDS-PAGE and then transferred in the PVDF blotting membrane. Rabbit sera were diluted in 0.5% skim milk (1:1,000) and then used as a primary antibody. A sheep anti-rabbit IgG antibody conjugated with HRP (diluted 1:10,000) was used as a secondary antibody. M, protein marker, Lane 1, purified rtHlATAQ; Lane 2, rProS2. Arrows indicate expected sizes of proteins.

Table S2. Effects of rHlATAQ vaccination on *H. longicornis* infestation in rabbits

Groups	Descriptive statistics	Blood feeding period (days)	Body weight of engorged ticks (mg)	Egg mass (mg)	Egg mass/body weight (%)	Pre-oviposition period (days)	Egg hatching period (days)
rHlATAQ vaccination							
	No of ticks	30	30	29[a]	29[a]	29[a]	29[a]
	Mean	8.1	222.22	113.34	49.29	5.59	35.14
	SD	0.5	25.69	15.3	10.38	0.63	1.77
	Q1	5	211.61	105	49.54	5	34
	Q2	6	222.68	114.9	51.86	6	35
	Q3	6	235.96	123.9	54.02	6	36
rPros2 vaccination							
	No of ticks	30	30	30	30	30	27[b]
	Mean	7.2	175.28	92.5	52.01	5.53	32.89
	SD	0.7	54.92	38.8	13.5	0.82	3.59
	Q1	5	124.98	61.18	46.03	5	30
	Q2	5	188.83	98.9	53.89	5	34
	Q3	6	223.29	123.65	58.37	6	35
p-value		$p < 0.01$	$p < 0.01$	$p < 0.01$	$p > 0.05$	$p < 0.01$	$p < 0.01$

Each group consisted of one rabbit. Each rabbit was infested with 30 females *H. longicornis* ticks. The rabbit vaccinated with rPros2 served as control. [a] One of the ticks fed on rHlATAQ-vaccinated rabbit did not lay eggs. [b] The eggs laid by 3 of the tick fed on rPros2-vaccinated rabbit did not hatch. The *p* value presents the significance of differences as computed using the Mann-Whitney U-test or the Tukey Honestly Significant Difference Test.
